# From Retina to Vasculature: Oxidative Stress as a Common Mechanistic Link Between Age-Related Macular Degeneration and Cardiovascular Disease

**DOI:** 10.3390/antiox15091140

**Published:** 2026-09-09

**Authors:** Jan Krekora, Jarosław Drożdż, Janusz Blasiak, Kai Kaarniranta

**Affiliations:** 1 2nd Department of Cardiology, Medical University of Lodz, 92-213 Lodz, Poland; ejkrekora@op.pl (J.K.); jaroslaw.drozdz@umed.lodz.pl (J.D.); 2 Faculty of Medicine, Mazovian University in Plock, 09-240 Plock, Poland; 3 Department of Ophthalmology, University of Eastern Finland, 70210 Kuopio, Finland; 4 Department of Ophthalmology, Kuopio University Hospital, 70210 Kuopio, Finland

**Keywords:** age-related macular degeneration, cardiovascular disease, oxidative stress, reactive oxygen and nitrogen species, mitochondrial dysfunction, inflammation, complement system, cellular senescence, biomarkers, aging

## Abstract

Increasing epidemiological and experimental evidence suggests that age-related macular degeneration (AMD) and cardiovascular disease (CVD) share multiple pathogenic mechanisms. Among these, oxidative stress has emerged as one of the most plausible links connecting retinal degeneration and cardiovascular pathology. Excessive production of reactive oxygen and nitrogen species, combined with declining antioxidant defenses, contributes to lipid peroxidation, mitochondrial dysfunction, chronic inflammation, complement activation, cellular senescence, and impaired cellular stress responses in both the retina and the vascular system. Notably, drusen (AMD) and atherosclerotic plaques (CVD) share several molecular constituents. Therefore, AMD and CVD may represent tissue-specific manifestations of broader age-related disturbances in redox homeostasis and inflammatory regulation. Nevertheless, the coexistence of AMD and CVD is incomplete, suggesting that genetic susceptibility, tissue-specific responses to oxidative stress, biological aging, and mechanisms of cellular resilience influence disease expression. In this review, we summarize current evidence linking AMD and CVD, examine oxidative stress-driven molecular pathways common to both disorders, discuss emerging biomarkers and therapeutic targets, and highlight important unresolved questions regarding disease heterogeneity and causal relationships. A better understanding of the shared mechanisms underlying AMD and CVD may facilitate the development of integrated preventive strategies, improved risk stratification, and more personalized therapeutic approaches for age-related diseases.

## 1. Introduction

Age-related macular degeneration (AMD) and cardiovascular disease (CVD) are among the leading causes of morbidity and disability worldwide, particularly in aging populations [1]. CVD is used here as an umbrella term encompassing coronary artery disease, stroke, and other vascular disorders. AMD is one of the leading causes of irreversible vision loss among older adults globally, although its burden is likely underestimated in low- and middle-income regions, while CVD remains the foremost cause of mortality globally [2,3]. Despite affecting distinct organ systems—the retina and the cardiovascular system-these conditions share some risk factors and pathological features and are underpinned by some shared mechanisms [4]. This suggests that AMD and CVD may not be entirely independent entities, but rather different manifestations of systemic pathological mechanisms that converge at both molecular and cellular levels.

Although it is difficult to find a condition without oxidative stress in its pathogenesis, AMD and CVD are both persuasively related to oxidative stress, understood as a state characterized by an imbalance between the production of reactive oxygen and nitrogen species (ROS) and the capacity of antioxidant defense systems to neutralize them [5,6]. Oxidative stress plays a well-established role in the pathogenesis of both AMD and CVD. In the retina, a tissue characterized by high metabolic activity, abundant polyunsaturated lipids, and chronic exposure to light, oxidative stress contributes to retinal pigment epithelium (RPE) dysfunction, photoreceptor degeneration, and the accumulation of extracellular deposits known as drusen [7]. In parallel, oxidative stress is a central driver of vascular pathology, promoting endothelial dysfunction, lipid oxidation, and the development of atherosclerotic plaques in CVD [8].

Beyond mechanistic parallels, AMD and CVD share several major risk factors, including aging, smoking, hypertension, dyslipidemia, and chronic inflammation [9]. Many of these are closely linked to increased oxidative burden, and this convergence supports the idea that oxidative stress may be a common upstream mediator rather than a coincidence. Furthermore, overlapping molecular pathways, including lipid peroxidation, complement activation, and mitochondrial dysfunction, have been implicated in both diseases, suggesting a common pathogenic framework [10,11]. Epidemiological studies have also explored associations between AMD and cardiovascular outcomes, although findings have been heterogeneous and sometimes conflicting, highlighting the complexity of this relationship [12].

Despite growing interest in oxidative stress as a unifying mechanism, important questions remain regarding its precise role in linking AMD and CVD. Specifically, it is unclear whether oxidative stress acts as a primary causal driver, a downstream consequence of disease processes, or a component of a broader network of interacting pathways [5,13]. Moreover, therapeutic strategies targeting oxidative stress in AMD and CVD have yielded mixed results, underscoring the need for a more nuanced understanding of its role in disease progression [14,15].

In this narrative review, we examine the current evidence supporting oxidative stress as a shared mechanistic link between AMD and CVD. We integrate findings from epidemiology, molecular biology, and clinical studies to explore common pathways and risk factors, evaluate the strengths and limitations of existing data, and discuss potential implications for prevention and therapy. By providing a comprehensive and critical overview, this work aims to clarify the extent to which oxidative stress contributes to the interplay between these two major age-related diseases.

This article was designed as a structured narrative and perspective review rather than a systematic review. The objective was not to comprehensively identify and quantitatively synthesize all available evidence, but to critically discuss and integrate current knowledge regarding the role of oxidative stress as a potential mechanistic link between AMD and CVD. Consequently, formal systematic review procedures, including PRISMA-guided study selection, predefined inclusion and exclusion criteria, and formal risk-of-bias assessment, were not performed. To enhance transparency and reproducibility, a structured literature search and qualitative critical appraisal of the retrieved evidence were conducted. A literature search was performed across databases such as PubMed, Embase, Scopus, Google Scholar, ScienceDirect, and the Cochrane Library. The focus was on publications from 2017 to 2026, with earlier foundational studies included when relevant. Search terms combined with Boolean operators included: “oxidative stress” AND (“AMD” OR “CVD”); “oxidative stress” AND (“retina” OR “heart”); “mitochondria” AND (“AMD” OR “CVD”); “inflammation” AND (“AMD” OR “CVD”); “complement” AND (“AMD” OR “CVD”); “senescence” AND (“AMD” OR “CVD”). No language restrictions were set. All publication types, such as original research (human, animal, and in vitro), systematic reviews, narrative reviews, and meta-analyses, were considered.

## 2. Epidemiological Links Between AMD and CVD

Clinically, advanced AMD is classified as either dry (non-exudative, atrophic; ICD code 35.30) or neovascular AMD (wet, exudative; ICD code 35.31) [16].

**Figure 1:**
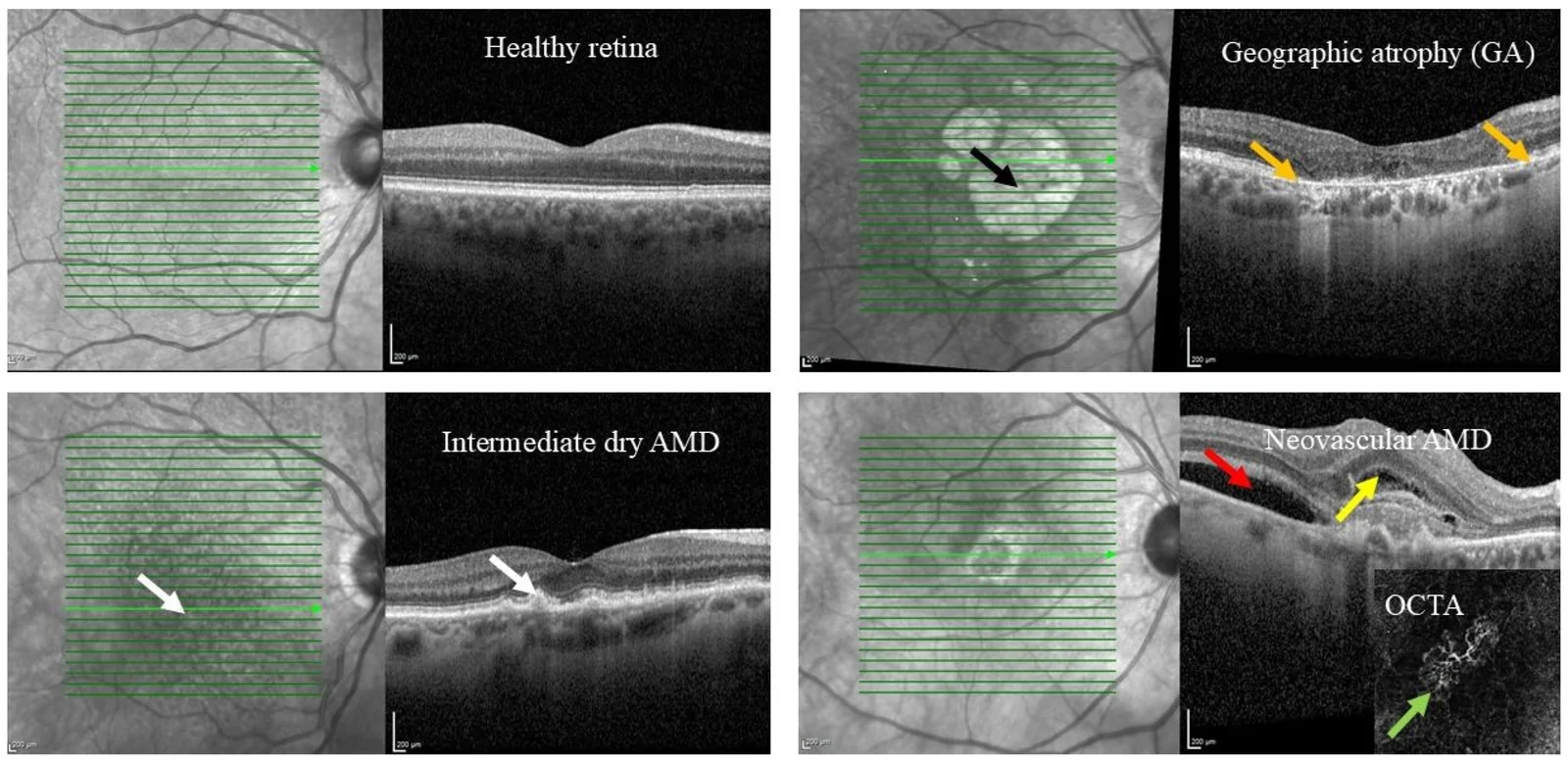
Optical coherence tomography (OCT) images from healthy retina and different phenotypes of age-related macular degeneration (AMD). For neovascular AMD, OCT angiography (OCTA) indicates active diagnostic neovascular membranes (green arrow). The white arrow stands for drusen, the black arrow for GA, the orange arrows for the edges of GA, the red arrow for subretinal fluid, and the yellow arrow for intraretinal fluid. Images were retrieved from the Kuopio University Hospital Imaging Database (courtesy of Kai Kaarniranta).

Neovascular AMD always follows the early stages of AMD, which are marked by the formation of drusen, extracellular deposits made up of lipids and proteins [17]. The intermediate stage of AMD is characterized by pigmentation irregularities in the retinal pigment epithelium (RPE). The late form of dry AMD, known as geographic atrophy (GA), is linked to the death of photoreceptors and the atrophy of supporting retinal pigment epithelium (RPE) cells, as well as the choriocapillaris, which may result in vision loss [18] (Figure 1).

Both AMD and CVD increase markedly with age, impose substantial healthcare burdens, and share several established risk factors, as mentioned in the previous section. These parallels implicate addressing the question of whether AMD and CVD represent distinct manifestations of a shared systemic pathological process or merely coexist due to overlapping demographic and clinical characteristics. While numerous epidemiological studies have reported associations between AMD and various cardiovascular outcomes, including coronary artery disease, myocardial infarction, stroke, and cardiovascular mortality, the overall evidence remains complex and at times contradictory.

The global prevalence of AMD is expected to increase substantially as populations age, making it an increasingly important public health concern. Similarly, CVD remains the leading cause of mortality globally, accounting for a significant proportion of deaths and disability among older individuals.

The coexistence of AMD and CVD has been reported in numerous population-based studies [4]. Individuals diagnosed with AMD frequently exhibit a higher prevalence of hypertension, atherosclerosis, dyslipidemia, and ischemic heart disease compared with age-matched controls [19]. Conversely, retinal changes characteristic of AMD appear more frequently among patients with established cardiovascular risk factors [1]. Such observations have supported the hypothesis that AMD may reflect not only localized retinal pathology but also broader systemic vascular dysfunction [20].

Particular attention has been paid to the association between late AMD and manifestations of atherosclerotic disease [21]. Several studies have demonstrated a higher prevalence of coronary artery disease, carotid artery abnormalities, and cerebrovascular disease in individuals with advanced AMD [1,22,23]. Although these findings do not establish causality, they suggest that the retinal and cardiovascular systems may share susceptibility to common biological stressors. Longitudinal cohort studies have provided valuable insights into the temporal relationship between AMD and cardiovascular outcomes. As mentioned, several large population-based investigations have reported that AMD, particularly late-stage disease, is associated with an increased risk of cardiovascular events, including myocardial infarction and stroke. In some cohorts, AMD has also been linked to increased cardiovascular and all-cause mortality [24].

Case–control studies have likewise demonstrated associations between AMD and atherosclerotic disease [25]. Patients with AMD often exhibit a greater burden of vascular risk factors and structural vascular abnormalities than control populations. Investigations utilizing carotid ultrasonography, coronary artery imaging, and measures of endothelial function have further supported the possibility of shared vascular pathology [26]. However, not all studies have reached similar conclusions. Some large cohort analyses have found only weak associations after adjustment for age and cardiovascular risk factors, while others have failed to identify any significant relationship between AMD and subsequent cardiovascular events [27,28]. Differences in study design, population characteristics, AMD classification systems, and endpoints likely contribute to these discrepancies.

Remarkably, as mentioned, stronger associations are often observed for advanced AMD than for early disease, suggesting that the severity of retinal pathology may be associated with an increased burden of systemic processes that also contribute to cardiovascular risk.”. This observation has led to speculation that progressive retinal degeneration may reflect cumulative exposure to systemic oxidative, inflammatory, and vascular insults [29,30].

Despite substantial research activity, the epidemiological relationship between AMD and CVD remains incompletely resolved. One major challenge is the strong influence of age, which is among the most important risk factors for both conditions. Even sophisticated statistical adjustments may not fully account for the effects of biological aging and age-related comorbidities. Smoking represents another significant confounder. As one of the strongest modifiable risk factors for AMD and a well-established contributor to CVD, smoking may partly explain observed associations between the two conditions. Similar concerns apply to hypertension, diabetes mellitus, obesity, and dyslipidemia.

Methodological heterogeneity further complicates interpretation. Studies differ in diagnostic criteria for AMD, definitions of cardiovascular outcomes, follow-up duration, and adjustment for confounding variables [31]. Moreover, many investigations rely on self-reported cardiovascular history, which may introduce misclassification bias [28].

Ethnic and geographic differences represent additional sources of variability. Most epidemiological data originate from North America, Europe, and other high-income regions, whereas data from low- and middle-income countries remain comparatively limited. Given potential differences in genetic background, environmental exposures, healthcare access, and disease detection rates, the generalizability of existing findings may be restricted. Finally, epidemiological associations alone cannot determine whether AMD directly contributes to cardiovascular pathology, whether CVD promotes retinal degeneration, or whether both conditions arise from shared pathogenic mechanisms.

Several hypotheses have been proposed to explain the observed epidemiological links between AMD and CVD. The most widely accepted explanation involves common environmental, metabolic, and biological risk factors that promote disease development in both the retina and the cardiovascular system [28,31].

Aging constitutes the principal non-modifiable risk factor for both diseases and is accompanied by progressive accumulation of oxidative damage, mitochondrial dysfunction, chronic low-grade inflammation, and impaired cellular repair mechanisms. Smoking further amplifies oxidative stress and vascular injury, contributing substantially to the development and progression of both AMD and CVD. Additional shared risk factors include hypertension, dyslipidemia, obesity, diabetes mellitus, and unhealthy dietary patterns. These conditions promote endothelial dysfunction, altered lipid metabolism, systemic inflammation, and increased production of reactive oxygen and nitrogen species (RONS). Importantly, many of these mechanisms are implicated in both retinal degeneration and atherosclerotic vascular disease [28,31,32].

The observation that AMD and CVD share common risk factors and biological pathways has led to growing interest in oxidative stress as a potential mechanistic bridge between the two conditions. Oxidative injury influences lipid oxidation, inflammatory signaling, complement activation, mitochondrial dysfunction, and cellular senescence, all of which contribute to pathological changes in both the retina and cardiovascular system [33,34,35,36]. Consequently, oxidative stress may provide a biologically plausible framework through which epidemiological associations can be interpreted, although causality remains to be established.

Epidemiological observations alone cannot determine whether AMD and CVD are causally related or simply share common risk factors. Nevertheless, the recurring association between these disorders has stimulated investigations into biological mechanisms operating in both the retina and the cardiovascular system. Among these, oxidative stress has emerged as one of the most plausible candidates and is discussed in the following sections.

## 3. Oxidative Stress as a Shared Pathogenic Framework Linking AMD and CVD

Oxidative stress is widely recognized as a central contributor to the pathogenesis of both AMD and CVD [37]. Although these disorders affect distinct tissues, they share several biological characteristics, and in both the retina and the vascular wall, excessive production of RONS and/or insufficient antioxidant defenses can disrupt cellular function, promote lipid and protein oxidation, trigger inflammatory responses, and accelerate tissue degeneration. Consequently, oxidative stress has emerged as one of the most plausible mechanistic links underlying the epidemiological and biological associations observed between AMD and CVD [12,38]. Shared molecular mechanisms in AMD and CVD involving oxidative damage, dysregulated lipid metabolism, mitochondrial dysfunction, chronic inflammation, and age-related loss of cellular resilience are tightly interconnected and form a self-amplifying network that promotes tissue degeneration in both the retina and the cardiovascular system [39]. The remarkable overlap of molecular signatures observed in AMD lesions and atherosclerotic plaques supports the concept that both disorders may represent tissue-specific manifestations of common pathogenic processes.

The retina is particularly vulnerable to oxidative damage owing to its exceptionally high metabolic activity, continuous exposure to visible light, and abundance of polyunsaturated fatty acids (PUFAs) that are prone to peroxidation. Furthermore, RPE cells are constantly challenged by the phagocytosis of photoreceptor outer segments (POS), a process associated with substantial oxidative burden [40]. As a result, maintenance of redox homeostasis is critical for retinal integrity.

Accumulating evidence suggests that oxidative stress contributes to AMD development from its earliest stages [41]. Excessive RONS production may damage lipids, proteins, mitochondrial DNA, and nuclear DNA within RPE cells, leading to impaired cellular function and reduced capacity for tissue repair [42]. Oxidative injury also promotes the accumulation of lipofuscin and other toxic by-products of cellular metabolism, further increasing susceptibility to retinal degeneration [43]. Thus, oxidative stress is not merely a consequence of retinal aging but appears to participate actively in multiple stages of AMD pathogenesis, linking mitochondrial dysfunction, chronic inflammation, complement activation, and retinal degeneration into a complex pathogenic network.

Oxidative stress plays an important role in the development and progression of CVD [44]. Under physiological conditions, RONS participate in intracellular signaling and vascular homeostasis, but excessive RONS production can overwhelm antioxidant defenses and promote vascular injury [45]. One of the earliest manifestations of oxidative stress in the cardiovascular system is endothelial dysfunction [46]. RONS reduce nitric oxide bioavailability, impair vasodilation, and promote a pro-inflammatory, pro-thrombotic vascular environment [47]. Endothelial dysfunction is widely regarded as a critical initiating event in atherogenesis and precedes the development of clinically evident cardiovascular disease. Oxidative stress represents a state of disrupted redox homeostasis in which the production of reactive oxygen and nitrogen species (RONS) exceeds the capacity of antioxidant and repair mechanisms, leading to oxidative modification of cellular macromolecules and altered intracellular signaling.

**Figure 2:**
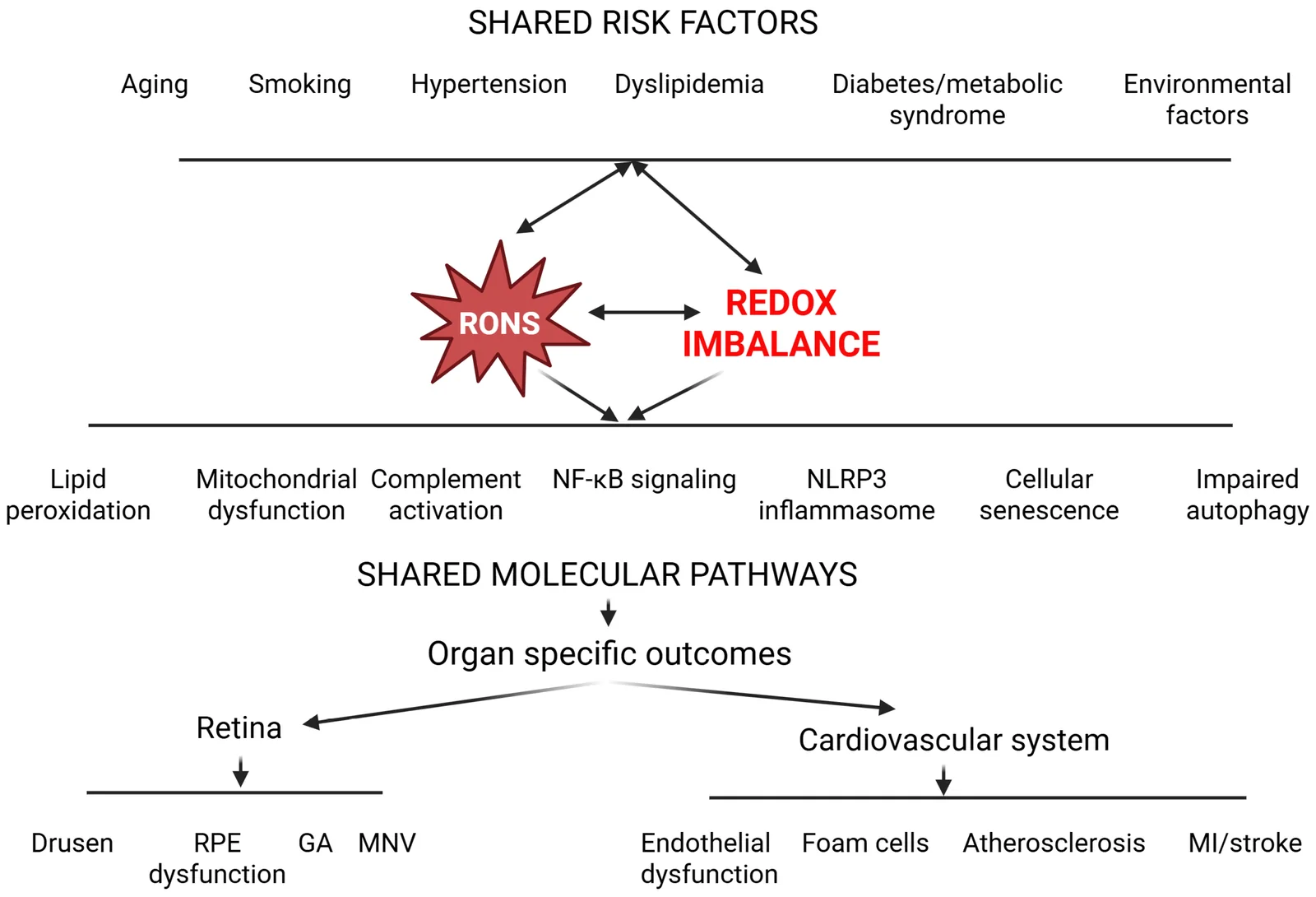
Oxidative stress as a central mechanistic hub linking age-related macular degeneration (AMD) and cardiovascular disease (CVD). Major environmental and clinical risk factors, including aging, smoking, hypertension, dyslipidemia, diabetes mellitus, obesity, and adverse lifestyle exposures, increase the production of reactive oxygen and nitrogen species (RONS) and/or impair antioxidant defenses. The resulting redox imbalance promotes a network of interconnected pathogenic processes, including lipid peroxidation, mitochondrial dysfunction, chronic inflammation, complement activation, cellular senescence, and impaired proteostatic and autophagic responses. These shared molecular pathways contribute to tissue-specific manifestations in the retina and cardiovascular system. In the retina, persistent oxidative stress promotes retinal pigment epithelium (RPE) dysfunction, drusen formation, photoreceptor degeneration, geographic atrophy (GA), and macular neovascularization (MNV). In the vasculature, similar mechanisms drive endothelial dysfunction, foam-cell formation, atherosclerotic plaque development, vascular remodeling, and ultimately clinical cardiovascular events such as myocardial infarction (MI) and stroke. Although AMD and CVD differ in their clinical presentation, both may represent organ-specific consequences of broader age-related disturbances in redox homeostasis and cellular stress adaptation. NF-κB, nuclear factor kappa B; NLRP3, nucleotide-binding oligomerization domain receptors, leucine-rich repeat and pyrin domain-containing protein 3. Created in BioRender. Błasiak, J. (2026) https://BioRender.com/p8k11ty (accessed 3 August 2026).

Importantly, oxidative stress rarely acts in isolation. Rather, it interacts closely with inflammation, dysregulated lipid metabolism, and aging-related cellular changes [48] (Figure 2). Collectively, these mechanisms drive the development of atherosclerotic lesions and increase the risk of adverse retinal events, including AMD and cardiovascular events, including myocardial infarction and stroke. These parallels suggest that AMD and CVD may represent tissue-specific manifestations of broader age-related disturbances in redox homeostasis. Although the retina and cardiovascular system differ substantially in structure and function, both appear vulnerable to the long-term cumulative effects of oxidative stress. This shared susceptibility provides a biologically plausible explanation for the epidemiological associations observed between AMD and CVD and supports the concept of oxidative stress as a common pathogenic denominator linking these conditions.

### 3.1. Oxidized Lipids and Lipoprotein Accumulation

Oxidative modification of lipids constitutes one of the most striking molecular similarities between AMD and CVD [49,50]. In the cardiovascular system, RONS generated by mitochondria, reduced nicotinamide adenine dinucleotide phosphate (NADPH) oxidases (NOX enzymes), xanthine oxidase, and uncoupled endothelial nitric oxide synthase (eNOS) induce the oxidation of low-density lipoproteins (LDL), resulting in the formation of oxidized LDL (oxLDL) [51]. OxLDL acts as a potent damage-associated molecular pattern (DAMP), stimulating endothelial activation, monocyte recruitment, foam-cell formation, and progression of atherosclerotic lesions [52].

Oxidative stress is also implicated in the formation of drusen, the extracellular deposits that characterize early AMD [53]. Many drusen components include oxidatively modified lipids, proteins, and complement-related molecules. These deposits can stimulate chronic local inflammation and activate the complement cascade, thereby linking oxidative injury with immune dysregulation [54]. A remarkably similar phenomenon occurs within the aging retina. RPE continuously processes POS rich in docosahexaenoic acid and other highly unsaturated lipids that are particularly susceptible to oxidative attack [55]. Lipid peroxidation generates reactive aldehydes, including 4-hydroxynonenal (4-HNE), malondialdehyde (MDA), and carboxyethylpyrrole (CEP), which accumulate in retinal tissues and have been detected within drusen [56]. In advanced AMD, persistent oxidative stress may contribute to geographic atrophy by promoting progressive loss of the retinal pigment epithelium and photoreceptors, while also facilitating neovascular AMD by activating proangiogenic pathways, including increased vascular endothelial growth factor (VEGF) signaling [57,58].

**Figure 3:**
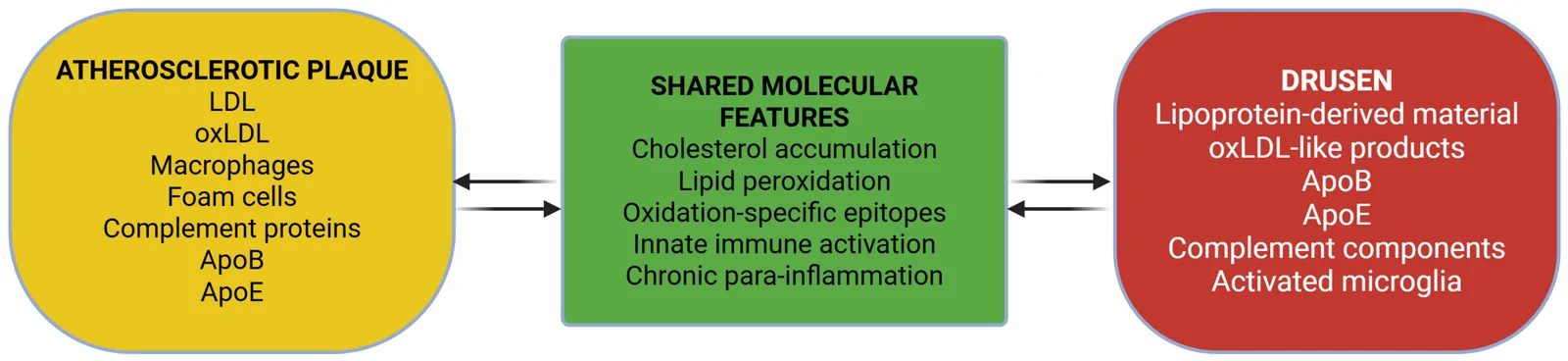
Comparison of the molecular composition and pathogenic consequences of extracellular lipid-rich deposits in age-related macular degeneration (AMD) and cardiovascular disease (CVD). In the arterial wall, oxidative modification of low-density lipoproteins (LDL) generates oxidized LDL (oxLDL), which promotes endothelial activation, recruitment of inflammatory cells, foam-cell formation, and progression of atherosclerotic plaques. In the retina, oxidative damage to lipid-rich material derived from photoreceptor outer segments and lipoprotein-like particles leads to the accumulation of oxidized lipids and proteins within Bruch’s membrane and drusen. Despite arising in distinct tissues, drusen and atherosclerotic plaques share numerous molecular constituents, including cholesterol, apolipoprotein B (ApoB), apolipoprotein E (ApoE), complement proteins, vitronectin, and oxidation-specific epitopes. These modified molecules function as damage-associated molecular patterns (DAMPs), activate scavenger receptors such as CD36 and scavenger receptor class A (SR-A), and promote chronic innate immune activation. The resulting para-inflammatory microenvironment contributes to progressive retinal degeneration in AMD and vascular injury in CVD. The shared molecular architecture of drusen and atherosclerotic plaques supports the concept that disturbed lipid homeostasis and oxidative lipid modification represent common pathogenic pathways linking retinal and cardiovascular disease. The narrowing of the diagram emphasizes oxidative stress as the principal point of convergence through which diverse risk factors influence a limited number of shared molecular pathways that subsequently diverge into distinct retinal and cardiovascular phenotypes. Created in BioRender. Błasiak, J. (2026) https://BioRender.com/llx3ecm (accessed 4 August 2026).

Importantly, drusen and atherosclerotic plaques share several molecular constituents, including cholesterol, apolipoprotein B (ApoB), apolipoprotein E (ApoE), vitronectin, complement components, and oxidation-specific epitopes [59] (Figure 3). Oxidatively modified lipoproteins can activate macrophages and microglia through scavenger receptors such as cluster of differentiation 36 (CD36) and scavenger receptor class A (SR-A), initiating inflammatory responses in both retinal and vascular tissues [60,61]. The concept of “para-inflammatory” lipid accumulation therefore provides a compelling molecular framework linking retinal degeneration and atherosclerotic vascular disease [62,63].

### 3.2. Mitochondrial Dysfunction

Mitochondrial dysfunction has emerged as a central driver of age-related diseases and may represent one of the most important mechanistic connections between AMD and CVD [64]. Mitochondria serve not only as the primary source of cellular ATP but also as critical regulators of redox signaling, apoptosis, calcium homeostasis, and innate immunity.

In AMD, mitochondrial DNA (mtDNA) damage accumulates progressively in RPE cells with age [57]. Because mtDNA lacks protective histones and possesses relatively limited repair capacity, it is particularly vulnerable to oxidative injury. Structural abnormalities of retinal mitochondria, including mitochondrial swelling, loss or disruption of cristae, fragmentation of the mitochondrial network, reduced mitochondrial number and mass, accumulation of damaged mitochondrial DNA (mtDNA), and altered mitochondrial membrane architecture, have been described in patients with AMD [57,65]. Each of these abnormalities can contribute to impaired electron transport chain (ETC) activity and reduced oxidative phosphorylation. These changes enhance electron leakage from respiratory complexes I and III, increasing superoxide production and further exacerbating oxidative stress.

Similar mechanisms have been identified in cardiovascular pathology [66]. Mitochondrial dysfunction in endothelial cells and vascular smooth muscle cells promotes excessive RONS generation, activation of redox-sensitive signaling pathways, and cellular senescence. In cardiomyocytes, mitochondrial impairment contributes to energetic failure, contractile dysfunction, and cell death [67].

**Figure 4:**
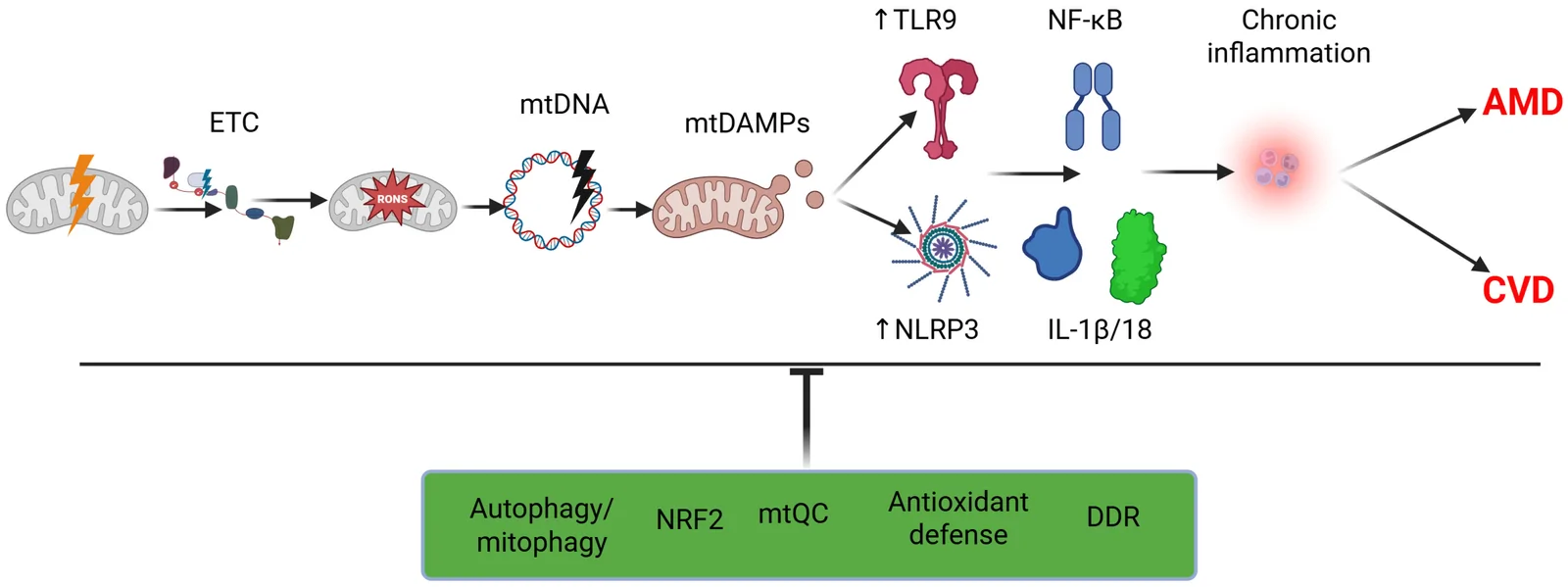
Mitochondrial dysfunction as a central link between oxidative stress and chronic inflammation in age-related macular degeneration (AMD) and cardiovascular disease (CVD). Oxidative stress impairs electron transport chain (ETC) function, increasing reactive oxygen and nitrogen species (RONS) production and promoting mitochondrial DNA (mtDNA), lipid, and protein damage. Damaged mitochondria release mitochondrial damage-associated molecular patterns (mtDAMPs), including mtDNA and cardiolipin, which activate Toll-like receptor 9 (TLR9) and the nucleotide-binding oligomerization domain receptor, leucine-rich repeat and pyrin domain-containing protein 3 (NLRP3) inflammasome. Subsequent activation of nuclear factor kappa B (NF-κB)-dependent signaling and pro-inflammatory cytokine production, represented here by interleukins IL-1α and IL-18, sustains chronic inflammation and tissue degeneration in both retinal and cardiovascular tissues. Counteracting mechanisms include nuclear factor erythroid 2-related factor 2 (NRF2)-dependent stress responses, DNA damage response (DDR) pathways, autophagy/mitophagy, and mitochondrial quality control (mtQC), which collectively maintain mitochondrial integrity, limit inflammation, and preserve cellular homeostasis. Age-related decline of these protective systems may increase susceptibility to both AMD and CVD. Created in BioRender. Błasiak, J. (2026) https://BioRender.com/427xqcd (accessed 4 August 2026).

A particularly important molecular link between AMD and CVD may be the release of mitochondrial DAMPs, including mtDNA and cardiolipin, which activate innate immune receptors such as Toll-like receptor 9 (TLR9) and the nucleotide-binding oligomerization domain receptors, leucine-rich repeat and pyrin domain-containing protein 3 (NLRP3) inflammasome [68,69] (Figure 4). Thus, mitochondrial injury may act as a bridge connecting oxidative stress to chronic inflammation in both AMD and CVD.

### 3.3. Inflammation and Complement Activation

Chronic inflammation is now recognized as a major pathogenic component of both AMD and CVD [1]. Oxidative damage generates modified proteins and lipids that the innate immune system perceives as danger signals, triggering persistent inflammatory responses.

In AMD, genetic studies have firmly established a role for the complement system [70]. Polymorphisms in genes encoding complement factor H (CFH), complement factor I (CFI), complement component 3 (C3), and complement factor B (CFB) significantly influence AMD susceptibility [71]. Oxidation-specific epitopes generated by lipid peroxidation bind complement regulators and may alter normal complement control, thereby promoting chronic inflammation within the subretinal space. Complement activation products such as C3a and C5a are frequently detected in drusen and can stimulate microglial activation, leukocyte recruitment, and vascular endothelial growth factor (VEGF) production [72].

Likewise, complement activation has increasingly been implicated in atherosclerosis [73]. OxLDL and apoptotic cellular debris activate complement pathways within the vascular wall, contributing to endothelial dysfunction and plaque progression. Beyond complement, both AMD and CVD exhibit activation of nuclear factor of kappa light chain enhancer of B cells (NF-κB)-dependent inflammatory signaling, increased expression of interleukin-1β (IL-1β), interleukin-6 (IL-6), and tumor necrosis factor-α (TNF-α), and upregulation of NLRP3 inflammasome components [30,74].

These observations suggest that oxidative stress and innate immune activation form a common pathogenic axis in AMD and CVD.

### 3.4. Cellular Senescence and Aging

Age is the strongest risk factor for both AMD and CVD, highlighting the importance of fundamental aging mechanisms in disease development. Cellular senescence is increasingly viewed as a central process linking biological aging to chronic degenerative disorders [75]. Senescent cells undergo irreversible cell-cycle arrest but remain metabolically active. Importantly, they acquire a senescence-associated secretory phenotype (SASP), characterized by the release of inflammatory cytokines, chemokines, growth factors, matrix metalloproteinases, and RONS [76]. SASP creates a chronic pro-inflammatory microenvironment that can propagate senescence to neighboring cells.

In AMD, senescence of RPE cells impairs phagocytosis, autophagy, and mitochondrial quality control (mtQC), promoting accumulation of damaged proteins and lipids [34]. Senescent RPE cells exhibit increased expression of cyclin-dependent kinase inhibitor 2A (CDKN2A, p16^INK4a^), cyclin-dependent kinase inhibitor 1A (CDKN1A, p21^CIP1)^, and tumor suppressor protein p53, accompanied by enhanced inflammatory signaling [77]. In the cardiovascular system, senescence affects endothelial cells, vascular smooth muscle cells, fibroblasts, and immune cells [78]. These changes contribute to arterial stiffness, endothelial dysfunction, vascular calcification, and chronic inflammation.

Oxidative stress is both a trigger and consequence of senescence, generating a self-perpetuating cycle of redox imbalance and tissue degeneration. Consequently, cellular senescence may represent a fundamental biological process underlying both retinal and vascular aging.

### 3.5. Impaired Redox Homeostasis as a Systemic Phenotype

Disease-related oxidative stress has been regarded as a local pathological process, but some evidence suggests that age-related disorders may arise from systemic deterioration of redox homeostasis across multiple tissues [79]. Several protective pathways that maintain cellular redox balance decline with age. These include reduced activity of cellular antioxidant systems and impaired activation of NRF2 [80]. NRF2 normally coordinates the expression of hundreds of cytoprotective genes involved in antioxidant defense, detoxification, and cellular stress adaptation [81]. Decreased NRF2 signaling has been implicated in both retinal degeneration and vascular disease [82,83].

In parallel, age-related impairment of autophagy and mitophagy reduces the capacity to eliminate damaged mitochondria and oxidized macromolecules. The resulting accumulation of dysfunctional organelles further increases RONS production and inflammatory signaling. The contribution of impaired autophagy to AMD and CVD has been extensively investigated, but in both diseases, some outstanding questions remain to be addressed to draw a definitive conclusion [84,85]. The primary reason for this ambiguity is the dual nature of autophagy, which may have either pro-life or pro-death properties in cellular homeostasis [86].

This systemic perspective offers a reasonable explanation for the common regulatory pathway in AMD and CVD. Rather than representing independent diseases connected by a single pathway, both may instead emerge from a broader failure of cellular homeostatic mechanisms that maintain redox equilibrium and tissue resilience throughout life. In this context, AMD could be viewed not only as a retinal disease but also as a visible manifestation of systemic biological processes that contribute to cardiovascular aging and pathology.

### 3.6. Common Risk Factors Converge on Shared Pathogenic Pathways

The remarkable overlap in the biological mechanisms underlying AMD and CVD may help explain why both disorders share a number of major risk factors, including aging, smoking, hypertension, dyslipidemia, obesity, and diabetes mellitus. Although diverse in their clinical manifestations, these exposures and conditions converge on a limited set of interconnected molecular pathways involving oxidative stress, mitochondrial dysfunction, chronic inflammation, impaired proteostasis, and cellular senescence. Consequently, they may promote pathological changes in both the retina and the cardiovascular system despite their distinct anatomical and functional characteristics.

Among these factors, aging is particularly important because it influences virtually all mechanisms discussed in this review. Progressive accumulation of oxidatively modified macromolecules, declining mitochondrial quality control, impaired autophagy, and reduced capacity to mount adaptive stress responses collectively increase tissue vulnerability [87]. Similarly, tobacco smoke, hyperglycemia, oxidized lipoproteins, and hypertension-associated activation of the renin-angiotensin system all enhance RONS generation and amplify inflammatory signaling pathways, including activation of NF-κB and the NLRP3 inflammasome [88]. These processes promote the formation of oxidation-specific epitopes, complement activation, and chronic para-inflammatory responses that are characteristic of both AMD and atherosclerotic CVD.

Importantly, the convergence of multiple risk factors on a common molecular network may explain why epidemiological associations between AMD and CVD are often strongest in advanced disease stages [26,27]. Rather than acting through separate mechanisms, individual risk factors may exert additive or synergistic effects on oxidative stress-driven pathways, progressively overwhelming cellular defense systems. From this perspective, AMD and CVD may be viewed not merely as coexisting age-related disorders but as tissue-specific outcomes of cumulative systemic disturbances in redox homeostasis, inflammatory regulation, and stress adaptation.

Taken together, the molecular pathways shared by AMD and CVD suggest that common risk factors do not operate as independent disease determinants but rather as modulators of a unified pathogenic network centered on oxidative stress, chronic inflammation, and age-related loss of cellular resilience. This concept provides a biologically plausible framework for understanding the epidemiological association between these disorders and may facilitate the identification of biomarkers and therapeutic targets relevant to both conditions.

## 4. Why Do Only Certain Individuals Develop Both Diseases?

Despite substantial epidemiological, molecular, and pathological overlap between AMD and CVD, the coexistence of these conditions is not common. Many individuals with advanced AMD never develop clinically apparent cardiovascular disease, while numerous patients with severe atherosclerosis show no evidence of retinal degeneration. This apparent discrepancy suggests that shared pathogenic mechanisms alone are insufficient to determine disease manifestation. Rather, disease development likely depends on complex interactions among genetic susceptibility, environmental exposures, tissue-specific responses to oxidative stress, and the intrinsic capacity of cells to maintain homeostasis during aging. Understanding why only a subset of individuals develop both diseases with a large spectrum of severity levels may provide important insights into the heterogeneity of age-related disorders and help identify biomarkers that distinguish vulnerability from resilience.

### 4.1. Genetic Susceptibility

Genetic factors are among the most likely explanations for the variable relationship between AMD and CVD. While both diseases are influenced by multiple genetic and environmental determinants, the relative contributions of individual pathways differ across tissues.

Among AMD-associated genes, variants in complement factor H (CFH), complement factor I (CFI), complement component 3 (C3), and the age-related maculopathy susceptibility 2/high-temperature requirement A serine peptidase 1 (ARMS2/HTRA1) locus have consistently demonstrated strong associations with disease risk [89]. Many of these genes are involved in innate immunity, complement regulation, and responses to oxidative stress. Their effects may be particularly pronounced within the highly specialized microenvironment of the retina, where complement activity requires tight regulation to maintain tissue integrity [90]. Conversely, CVD is strongly influenced by genes regulating lipid metabolism, vascular function, thrombosis, and blood pressure control, including variants affecting apolipoprotein E (APOE), low-density lipoprotein receptor (LDLR), proprotein convertase subtilisin/kexin type 9 (PCSK9), and genes within the renin-angiotensin system [91]. Although certain genetic determinants may contribute to both disorders, their effects are unlikely to be identical across tissues.

An emerging concept is that genetic variation influences not only susceptibility to disease but also the capacity to tolerate oxidative stress [92]. Polymorphisms affecting antioxidant enzymes, mitochondrial function, complement regulation, inflammatory signaling, and cellular repair mechanisms may determine whether a given level of oxidative burden leads predominantly to retinal degeneration, vascular pathology, or both.

### 4.2. Systemic Versus Local Oxidative Stress

The relationship between systemic oxidative stress and tissue-specific injury remains incompletely understood. While markers of oxidative damage can often be detected in the circulation, their presence does not necessarily predict which organs will ultimately develop pathology.

As mentioned, the retina possesses unique metabolic characteristics that may amplify the consequences of oxidative stress. High oxygen consumption, continuous exposure to visible light, intensive mitochondrial activity, and the abundance of PUFAs create an environment particularly vulnerable to lipid peroxidation and oxidative injury. Consequently, even modest systemic disturbances may have disproportionately large effects on retinal tissues. In contrast, the cardiovascular system is exposed to distinct stressors, including hemodynamic forces, endothelial injury, altered lipid transport, and chronic vascular inflammation [93]. Thus, although oxidative stress is a common denominator, the mechanisms through which it is generated and translated into disease may differ substantially between tissues.

These observations suggest that retinal and vascular pathology may arise from a combination of systemic oxidative burden and local factors that determine tissue-specific susceptibility.

### 4.3. Tissue-Specific Responses to Shared Insults

Aging, smoking, hypertension, dyslipidemia, and metabolic dysfunction expose multiple organs to similar molecular insults. Nevertheless, identical systemic challenges can produce markedly different pathological outcomes in different tissues and organs. One explanation of diversity is that RPE cells, photoreceptors, endothelial cells, vascular smooth muscle cells, and macrophages possess distinct metabolic requirements and stress-response programs [94]. Differences in mitochondrial dynamics, autophagic capacity, antioxidant defenses, and inflammatory signaling may determine how individual cell types respond to oxidative injury. As mentioned, RPE cells rely heavily on efficient POS degradation and maintenance of visual cycle metabolism. Impairment of these functions can result in lipofuscin accumulation, drusen formation, and retinal degeneration. In contrast, endothelial cells respond to oxidative stress primarily through alterations in nitric oxide signaling, vascular inflammation, and barrier function, ultimately promoting atherosclerotic lesion development [95].

The same molecular trigger may therefore initiate fundamentally different pathological cascades depending on cellular context. Such tissue-specific responses may help explain why shared risk factors and molecular pathways do not lead to identical clinical outcomes across even apparently identical individuals.

### 4.4. Resilience, Compensation, and Biological Aging

The development of age-related disease is increasingly viewed as a balance between damaging processes and protective mechanisms. Individuals differ considerably in their ability to maintain redox homeostasis, preserve mitochondrial quality, regulate inflammation, and repair molecular damage.

Protective pathways involving NRF2, autophagy, mitophagy, DNA repair systems, antioxidant enzymes, molecular chaperones and, to a smaller extent, low-molecular-weight antioxidants can partially counteract the harmful effects of oxidative stress. Likewise, efficient removal of damaged mitochondria and suppression of excessive inflammatory activation may delay the onset of both AMD and CVD despite the presence of significant risk factors.

**Figure 5:**
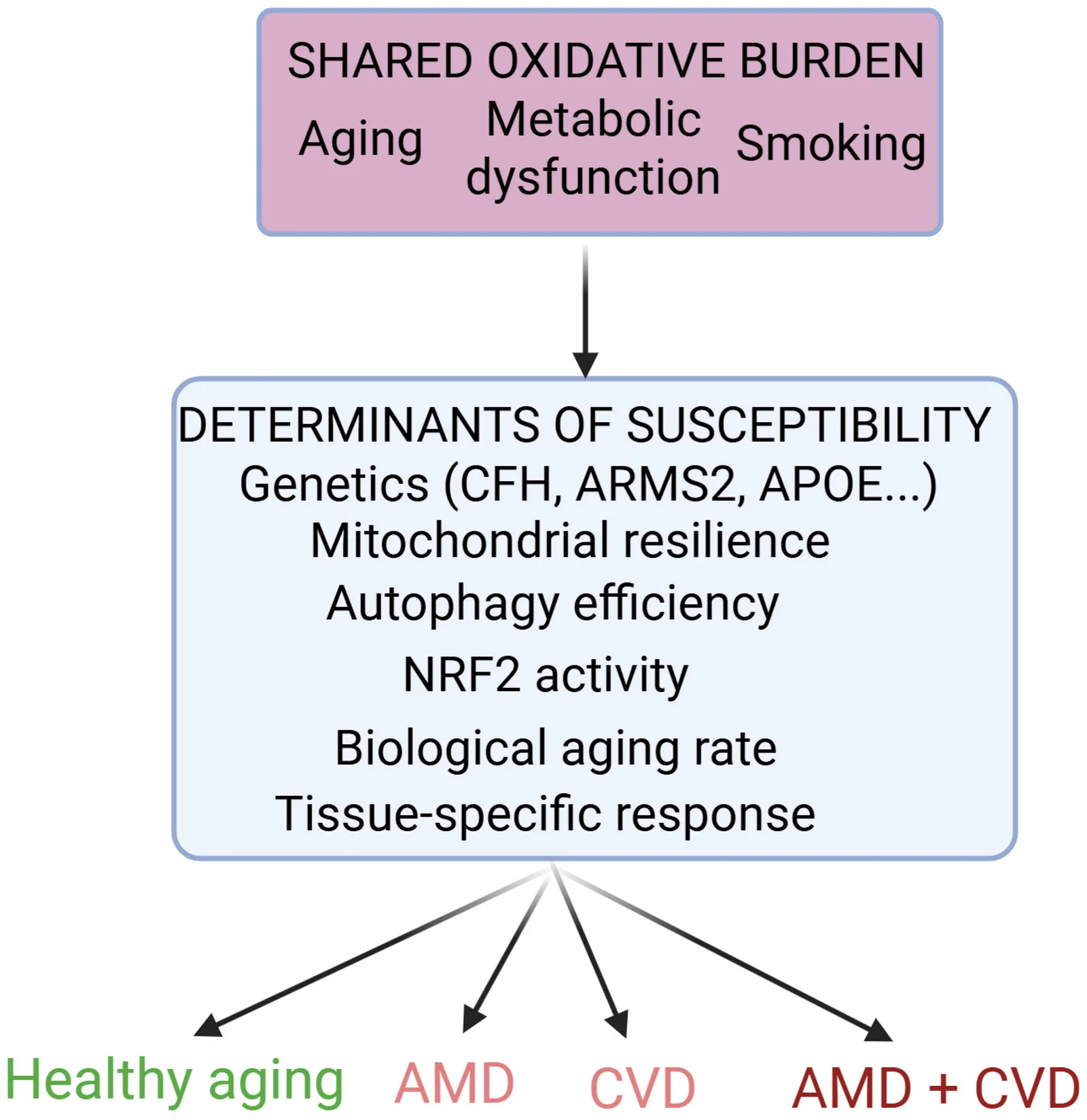
Proposed model explaining the incomplete overlap between age-related macular degeneration (AMD) and cardiovascular disease (CVD). Common environmental and clinical risk factors, including aging, smoking, hypertension, dyslipidemia, and metabolic dysfunction, increase systemic oxidative stress and promote activation of shared pathogenic pathways. However, exposure to similar oxidative and inflammatory burdens does not necessarily result in identical clinical outcomes. Genetic susceptibility, biological aging, mitochondrial resilience, DNA damage response, autophagy and mitophagy efficiency, nuclear factor erythroid 2-related factor 2 (NRF2)-dependent stress responses, and tissue-specific adaptive mechanisms may modulate individual vulnerability to disease. As a result, the same systemic stressors may lead predominantly to retinal degeneration (AMD), cardiovascular pathology (CVD), coexistence of both disorders, or successful maintenance of tissue homeostasis and healthy aging. CHF, complement factor H; ARMS2, the age-related maculopathy susceptibility 2; APOE, apolipoprotein E. Created in BioRender. Błasiak, J. (2026) https://BioRender.com/pi6b4vr (accessed 5 August 2026).

The concept of biological rather than chronological aging may be particularly relevant in this context. Individuals of the same chronological age may exhibit markedly different levels of oxidative damage, cellular senescence, mitochondrial dysfunction, and inflammatory activity. Consequently, susceptibility to AMD, CVD, or both may depend less on age itself than on the cumulative integrity of cellular maintenance systems (Figure 5).

### 4.5. AMD as a Potential Marker of Systemic Vulnerability

The coexistence of AMD and CVD in some individuals raises the possibility that AMD may, at least in certain cases, reflect broader systemic disturbances extending beyond the eye. Because retinal tissues can be visualized noninvasively, AMD has often been viewed as a potential indicator of underlying biological processes occurring elsewhere in the body. In extreme cases, advanced neovascular AMD may be associated with a higher mortality rate [96].

From this perspective, the retina may serve as a sensitive reporter of cumulative oxidative stress, chronic inflammation, and impaired stress adaptation. However, the extent to which AMD reflects generalized vascular aging rather than primarily localized retinal pathology remains uncertain. Current evidence suggests that AMD is neither a simple ocular manifestation of cardiovascular disease nor a universally reliable predictor of future cardiovascular events. Instead, AMD may identify a subset of individuals in whom systemic mechanisms related to oxidative stress and aging are particularly active. Future studies integrating retinal imaging, cardiovascular phenotyping, genomic analyses, and molecular biomarkers may clarify whether AMD can be used to identify patients at increased risk of broader age-related pathology.

In conclusion, the incomplete overlap between AMD and CVD highlights the complexity of age-related disease biology. Shared molecular pathways provide a common pathogenic framework, but genetic background, tissue-specific responses, and individual differences in resilience determine whether these mechanisms culminate in retinal degeneration, cardiovascular disease, or both. Understanding these determinants of vulnerability may ultimately prove as important as understanding the shared mechanisms themselves, offering new opportunities for risk stratification, early detection, and personalized intervention.

## 5. Biomarkers Relevant to Both AMD and CVD

The substantial overlap in the molecular mechanisms underlying AMD and CVD has stimulated interest in biomarkers of processes common to both diseases. A clinically useful biomarker could facilitate early detection, improve risk stratification, provide insights into disease progression, and identify individuals who may benefit from targeted preventive or therapeutic interventions. Although no single biomarker has yet demonstrated sufficient specificity or predictive value for routine clinical use in both conditions, a growing body of evidence suggests that markers of oxidative stress, inflammation, complement activation, mitochondrial dysfunction, and cellular aging may provide valuable information regarding the shared biological pathways linking AMD and CVD.

### 5.1. Biomarkers of Oxidative Damage

As oxidative stress occupies a central position in the pathogenesis of both AMD and CVD, numerous studies have investigated products of oxidative macromolecular damage as potential biomarkers. Lipid peroxidation products are among the most extensively studied indicators. Elevated concentrations of MDA, 4-HNE, F2-isoprostanes, and oxLDL have been reported in patients with cardiovascular disorders and have also been associated with AMD development and progression [97,98]. These molecules not only reflect oxidative injury but may actively participate in disease pathogenesis by generating oxidation-specific epitopes that trigger inflammatory responses and complement activation.

Increased levels of 8-hydroxy-2′-deoxyguanosine (8-OHdG), a marker of oxidative DNA modification, have been detected in both retinal tissues affected by AMD and in patients with cardiovascular disease [99,100]. Similarly, protein oxidation products, such as advanced oxidation protein products (AOPPs) and carbonylated proteins, may serve as indirect indicators of systemic oxidative burden.

Despite their biological relevance, many oxidative stress biomarkers exhibit considerable variability and are influenced by age, smoking status, diet, medication use, and comorbidities, which currently limits their clinical applicability.

### 5.2. Inflammatory and Complement-Related Biomarkers

Inflammation represents another important source of candidate biomarkers shared between AMD and CVD. Among circulating inflammatory markers, C-reactive protein (CRP) has been most extensively investigated. Elevated CRP concentrations have been associated with increased cardiovascular risk and have also been linked to AMD in several studies, supporting the concept that systemic low-grade inflammation contributes to both disorders [101,102]. Additional inflammatory mediators of interest include IL-1β, IL-6, TNF-α, monocyte chemoattractant protein-1 (MCP-1), and other cytokines involved in innate immune activation [30,74]. Increased concentrations of these molecules have been described in patients with AMD and various forms of CVD, although their specificity remains limited.

Particularly relevant to AMD are biomarkers reflecting complement activation. Elevated circulating levels of complement activation fragments, including C3a, C5a, and soluble C5b-9, have been associated with AMD, while complement dysregulation has also been implicated in atherosclerosis and vascular inflammation [103,104]. Consequently, complement-related biomarkers may serve as promising indicators of shared immune mechanisms in AMD and CVD.

### 5.3. Lipid-Related Biomarkers

Given the importance of lipid metabolism in both AMD and CVD, several lipid-related biomarkers have been explored as potential indicators of common pathogenic processes. Beyond conventional lipid measurements such as LDL cholesterol and HDL cholesterol, increasing attention has focused on oxidatively modified lipoproteins and apolipoproteins. Elevated oxLDL concentrations are associated with endothelial dysfunction and atherosclerosis and have likewise been linked to retinal degeneration [105,106]. Similarly, altered levels of ApoB and ApoE have been observed in both retinal and cardiovascular pathology [107,108].

Lipoprotein(a) (Lp(a)), a recognized cardiovascular risk factor, has also been investigated in relation to AMD, although available findings remain inconsistent [109,110]. Likewise, disturbances in cholesterol transport and reverse cholesterol transport pathways have emerged as potential contributors to both diseases [111,112].

These observations support the concept that lipid-related biomarkers may reflect a shared disturbance in lipid handling and oxidative modification rather than tissue-specific pathology alone.

### 5.4. Biomarkers of Mitochondrial Dysfunction and Cellular Aging

Circulating cell-free mitochondrial DNA (cf-mtDNA) has emerged as a potential indicator of mitochondrial injury and systemic inflammation in several pathologies [113]. Released from damaged cells, cf-mtDNA may function as a mitochondrial damage-associated molecular pattern (mtDAMP), activating TLR9 and the NLRP3 inflammasome [69]. Elevated cf-mtDNA concentrations have been reported in several cardiovascular conditions [114]. Although we cannot find a study demonstrating the involvement of cf-mtDNA in AMD pathology, strong evidence for the role of impaired mitochondrial quality control in AMD suggests that cf-mtDNA might be a marker of this disease [57].

Other potential biomarkers include indicators of impaired mitochondrial bioenergetics, altered mitochondrial DNA copy number, and molecules associated with defective mitophagy and autophagy [42,115]. Although these markers remain largely investigational, they are particularly attractive because they hold a central position within the shared mechanistic framework linking AMD and CVD.

Markers of biological aging and cellular senescence have also received increasing attention in AMD and CVD [34,116]. Telomere shortening, increased expression of p16^INK4a^, SASP factors, and epigenetic aging signatures may provide insights into an individual’s susceptibility to age-related degenerative disorders, as partly presented in Section 3. However, their clinical utility in AMD and CVD remains to be established.

### 5.5. Emerging Multi-Omics and Imaging Biomarkers

The complexity of AMD and CVD suggests that no single biomarker is likely to capture the full spectrum of disease-related processes. Consequently, increasing efforts are being directed toward integrated biomarker approaches that combine genomics, transcriptomics, proteomics, metabolomics, and lipidomics [117,118].

Particular interest has focused on oxidative lipid metabolites, complement-associated proteins, inflammatory signatures, and markers of mitochondrial function. Such approaches may facilitate identification of molecular endotypes that transcend traditional disease classifications and help explain why some individuals develop AMD, CVD, or both.

Retinal imaging may also provide a unique window into the biology of systemic disease. Advances in optical coherence tomography (OCT), OCT angiography, and quantitative retinal image analysis have raised the possibility that retinal structural and vascular changes could serve as surrogate markers of systemic vascular aging and cardiovascular risk [119]. Because the retina is the only part of the microvasculature that can be directly visualized noninvasively, retinal imaging may offer valuable opportunities for integrated assessment of ocular and cardiovascular health.

Current evidence suggests that AMD and CVD share a broad spectrum of biomarkers reflecting oxidative stress, lipid peroxidation, inflammation, complement activation, mitochondrial dysfunction, and biological aging. However, no individual marker has yet demonstrated sufficient sensitivity or specificity to serve as a universal indicator of both disorders. Future progress will likely depend on multimodal approaches integrating molecular biomarkers with genetic information and advanced retinal imaging. Such strategies may improve our understanding of the biological relationship between AMD and CVD and facilitate the development of personalized approaches to risk assessment and disease prevention.

## 6. Therapeutic Implications, Preventive Strategies, and Future Directions

The recognition that AMD and CVD share multiple pathogenic pathways has important therapeutic and preventive implications. Oxidative stress, mitochondrial dysfunction, lipid peroxidation, complement activation, chronic inflammation, and cellular senescence are increasingly viewed not only as mechanisms of disease but also as potential therapeutic targets. However, despite compelling biological evidence, successful translation into clinical interventions has proven challenging. This apparent discrepancy highlights the complexity of redox biology and the need for more precise therapeutic approaches. Future advances will likely depend on integrated strategies that address the interconnected molecular networks underlying both retinal and cardiovascular pathology, rather than targeting individual pathways in isolation.

### 6.1. Lessons from Antioxidant Therapy

The central role of oxidative stress in both AMD and CVD initially generated considerable attention for antioxidant-based interventions. However, clinical experiences have differed substantially between the two diseases.

In AMD, the strongest evidence originates from the Age-Related Eye Disease Studies (AREDS and AREDS2), which demonstrated that nutritional supplementation containing selected antioxidants and zinc can reduce the risk of progression from intermediate to advanced AMD in specific patient populations [120,121]. These findings provide proof of principle that modulation of oxidative stress may influence disease progression under appropriate conditions.

In contrast, large cardiovascular trials using antioxidant vitamins have generally produced disappointing or inconsistent results [122,123,124]. Although experimental studies consistently demonstrate beneficial effects of antioxidant interventions on vascular function and oxidative injury, these findings have not consistently translated into reductions in cardiovascular events. Several explanations have been proposed, including inadequate targeting of disease-relevant reactive species, poor bioavailability, treatment initiated too late in the disease process, and the physiological importance of RONS in normal cellular signaling.

Collectively, these observations suggest that generalized antioxidant supplementation is unlikely to provide a universal solution for oxidative stress-associated diseases. Future interventions will probably require greater specificity and a deeper understanding of disease stage, patient selection, and molecular targets.

### 6.2. Targeting Mitochondrial Dysfunction and Redox Signaling

Because mitochondria represent both a major source and target of oxidative stress, strategies aimed at preserving mitochondrial function in AMD and CVD are justified [57,125]. Potential approaches include mitochondrial-targeted antioxidants, compounds enhancing mitochondrial biogenesis, modulators of mitophagy, and interventions improving mtQC [98,126]. Experimental studies have demonstrated that such strategies may reduce oxidative damage, suppress inflammatory signaling, and improve cellular survival in both retinal and vascular tissues.

Particular attention has focused on therapies that restore physiological redox signaling rather than indiscriminately suppressing RONS. Activation of NRF2, also by natural compounds, represents a promising example [127]. As a master regulator of antioxidant defense, NRF2 controls the expression of numerous genes involved in detoxification, redox homeostasis, and cellular stress adaptation. Declining NRF2 activity has been implicated in both AMD and CVD, raising the possibility that pharmacological enhancement of endogenous cytoprotective pathways may offer greater benefits than conventional antioxidant supplementation [83,128].

### 6.3. Anti-Inflammatory and Complement-Oriented Approaches

The close interplay between oxidative stress and inflammation has stimulated considerable interest in therapies targeting innate immune pathways. In AMD, complement inhibition has emerged as a major therapeutic strategy, especially for geographic atrophy [129]. The approval of complement inhibitors (pegcetacoplan (C3 inhibitor), avacincaptad pegol (C5 inhibitor)) for geographic atrophy has strengthened the evidence implicating complement dysregulation in AMD pathogenesis, although their modest efficacy and potential adverse effects indicate that the complement system represents only one component of a broader pathogenic network [130,131].

Similarly, cardiovascular research increasingly recognizes inflammation as a therapeutic target. Clinical studies demonstrating improved cardiovascular outcomes following inhibition of IL-1β signaling have provided evidence that modulation of innate immune pathways can alter disease progression independently of conventional lipid-lowering approaches [132].

These developments support the concept that oxidative stress and inflammation should not be viewed as separate therapeutic targets but rather as components of an integrated pathogenic network. Future therapeutic strategies may therefore combine redox-modulating and immune-modulating interventions.

### 6.4. Lifestyle-Based Prevention and Systemic Risk Reduction

Despite advances in molecular therapeutics, lifestyle modification remains one of the most effective means of reducing the burden of both AMD and CVD. Smoking cessation is particularly important, given the strong association between tobacco exposure, oxidative stress, retinal degeneration, and vascular disease. Likewise, regular physical activity improves mitochondrial function, enhances antioxidant defenses, and reduces chronic inflammation.

Dietary patterns may also influence disease risk through their effects on oxidative stress and lipid metabolism. Diets rich in fruits, vegetables, fish, and other sources of antioxidants and omega-3 fatty acids have been associated with improved retinal and cardiovascular health [133,134]. Conversely, diets characterized by excessive consumption of saturated fats and ultra-processed foods may amplify oxidative injury and inflammatory responses. Importantly, these preventive measures target multiple pathogenic pathways simultaneously and may therefore provide broader benefits than therapies directed against a single molecular mechanism.

### 6.5. Precision Medicine and Biomarker-Guided Interventions

Due to the heterogeneous nature of AMD and CVD, their patients may differ substantially in the relative contribution of oxidative stress, complement dysregulation, mitochondrial dysfunction, lipid abnormalities, or chronic inflammation to disease development. This heterogeneity may partly explain the inconsistent results of several therapeutic interventions. A treatment targeting oxidative stress may be effective only in individuals exhibiting a predominantly oxidative-stress-driven disease phenotype. Consequently, future management strategies will likely rely increasingly on biomarker-guided patient stratification. Integration of genetic information, circulating biomarkers, retinal imaging, and cardiovascular phenotyping may allow identification of disease endotypes characterized by distinct molecular signatures. Such approaches could facilitate personalized prevention and treatment strategies while improving the efficiency of clinical trials.

Potential therapeutic implications for shared pathogenic pathways in AMD and CVD are summarized in Table 1.

## 7. Conclusions and Outstanding Questions

Accumulating epidemiological, molecular, and clinical evidence indicates that AMD and CVD share multiple pathogenic mechanisms. Among these, oxidative stress emerges as a particularly compelling link, acting at the intersection of mitochondrial dysfunction, dysregulated lipid metabolism, chronic inflammation, complement activation, cellular senescence, and impaired adaptive stress responses. Although the retina and cardiovascular system differ markedly in structure and physiological function, both appear vulnerable to the cumulative biological consequences of oxidative injury and age-related loss of cellular resilience.

The remarkable similarities between drusen and atherosclerotic plaques, the involvement of shared inflammatory and complement pathways, and the contribution of mitochondrial dysfunction to both retinal and vascular degeneration support the concept that AMD and CVD may represent tissue-specific manifestations of broader systemic disturbances in redox homeostasis. Nevertheless, available evidence does not support a simple causal relationship between these conditions. Rather, it suggests a complex network of interacting mechanisms whose relative contributions vary across individuals, tissues, and stages of disease.

One of the most intriguing observations is that only a subset of individuals develops both disorders despite exposure to similar environmental risk factors and activation of apparently common molecular pathways. This heterogeneity highlights the likely importance of genetic susceptibility, tissue-specific responses to oxidative stress, differences in biological aging, and the efficiency of protective mechanisms such as mitochondrial quality control, autophagy, and NRF2-dependent stress responses. Understanding these determinants of vulnerability and resilience may prove as important as identifying the mechanisms that drive disease itself.

Although antioxidant-based therapies have yielded mixed results, recent advances in complement inhibition, mitochondrial biology, biomarker exploitation, and precision medicine provide renewed opportunities for therapeutic innovation. Increasing recognition that oxidative stress participates in an integrated pathogenic network rather than acting as an isolated process may help explain previous translational failures and guide the development of more targeted, mechanism-based interventions.

Several important questions, however, remain unresolved. First, the precise contribution of oxidative stress to disease initiation and progression requires further clarification, particularly regarding its role as a primary driver versus a downstream amplifier of pathological processes. Second, the extent to which AMD reflects systemic vascular dysfunction or a broader phenotype of pathological aging remains uncertain. Third, reliable biomarkers capable of identifying individuals at risk for both AMD and CVD are still lacking. Finally, a better understanding of the molecular mechanisms underlying resistance to disease may reveal novel opportunities for prevention and healthy aging.

Future research should integrate longitudinal clinical studies with advanced molecular profiling, including genomics, transcriptomics, proteomics, metabolomics, and lipidomics. Combined with artificial intelligence-assisted analysis of retinal imaging and cardiovascular phenotyping, these approaches may help define biological endotypes that transcend conventional disease classifications. Such efforts could ultimately enable earlier identification of individuals at risk, improved patient stratification, and the development of personalized preventive and therapeutic strategies.

In conclusion, oxidative stress provides a biologically plausible and experimentally supported framework for understanding the relationship between AMD and CVD. While many aspects of this relationship remain incompletely understood, the convergence of epidemiological observations and molecular evidence suggests that studying these disorders together may yield insights extending beyond ophthalmology and cardiovascular medicine, contributing more broadly to our understanding of aging-related degenerative disease.

## Figures and Tables

**Table 1:** Shared pathogenic pathways linking age-related macular degeneration (AMD) and cardiovascular disease (CVD) and their potential therapeutic implications. Most currently available interventions target individual components of a complex network involving oxidative stress, mitochondrial dysfunction, dysregulated lipid metabolism, inflammation, complement activation, cellular senescence, and impaired autophagy. Future therapeutic strategies may benefit from integrated approaches aimed at restoring cellular resilience and maintaining redox homeostasis rather than suppressing isolated pathological processes.

Shared Pathway	Biological Consequence		Potential Therapeutic Approach	Major Challenges
	AMD	CVD		
Oxidative stress and redox imbalance	RPE *^a^* dysfunction, drusen formation, photoreceptor degeneration	Endothelial dysfunction, LDL oxidation, vascular injury	AREDS-type supplementation, NRF2 activators, redox-modulating compounds, lifestyle interventions	Limited efficacy of non-targeted antioxidants; physiological roles of RONS must be preserved
Lipid peroxidation and oxidized lipoproteins	Accumulation of oxidized lipids in Bruch’s membrane and drusen	Foam-cell formation and atherosclerosis	Lipid lowering, modulation of lipid metabolism, inhibition of oxidative lipid modification	Incomplete understanding of tissue-specific lipid pathways
Mitochondrial dysfunction	Impaired ATP production, RPE degeneration, increased oxidative stress	Endothelial dysfunction, cardiomyocyte injury, vascular aging	Mitochondrial-targeted antioxidants, stimulation of mitochondrial biogenesis, enhancement of mitophagy and mtQC	Limited clinical evidence and lack of validated biomarkers
Inflammation	Chronic retinal inflammation, microglial activation	Chronic vascular inflammation, plaque progression	Anti-inflammatory therapies, NF-κB pathway modulation, inflammasome-targeted interventions	Risk of systemic immunosuppression and variable patient responses
Complement dysregulation	Drusen formation, progression of geographic atrophy, neovascularization	Amplification of vascular inflammation and atherogenesis	Complement inhibitors (e.g., C3/C5-targeted therapies)	Long-term efficacy, optimal timing, patient selection
Cellular senescence	RPE dysfunction, impaired tissue repair, SASP-associated inflammation	Vascular aging, arterial stiffness, chronic inflammation	Senolytics, senomorphics, modulation of SASP	Safety and long-term consequences remain uncertain
Impaired autophagy and mitophagy	Accumulation of damaged proteins, lipids, and mitochondria	Defective mitochondrial turnover and vascular dysfunction	Pharmacological enhancement of autophagy and mitophagy	Dual protective and detrimental roles depending on disease stage
Declining cellular resilience	Reduced capacity to counter oxidative and metabolic stress	Increased susceptibility to vascular injury and aging	Lifestyle interventions, exercise, caloric balance, activation of endogenous stress-response pathways (NRF2, AMPK, sirtuins)	Lack of validated resilience biomarkers

*^a^* Abbreviations: AMPK, 5′AMP-activated protein kinase; AREDS, Age-Related Eye Disease Study; LDL, low-density lipoproteins; mtQC, mitochondrial quality control; NF-κB, nuclear factor kappa B; NRF2, nuclear factor erythroid 2-related factor 2; RPE, retinal pigment epithelium; SASP, senescence-associated secretory phenotype.

## Data Availability

No new data were created or analyzed in this study. Data sharing is not applicable to this article.
